# General practitioners' attitudes towards research in primary care: qualitative results of a cross sectional study

**DOI:** 10.1186/1471-2296-5-31

**Published:** 2004-12-21

**Authors:** Thomas Rosemann, Joachim Szecsenyi

**Affiliations:** 1 Department. of General Practice and Health Services Research, University of Heidelberg, Voßstr. 2, 69115 Heidelberg, Germany

## Abstract

**Background:**

Research in General Practice requires the participation of General practitioners (GPs). In Germany there is little tradition of research in this field, and GPs are not used to be participants in research. Little is known about German GPs attitudes towards research. Therefore the aim of our study was to assess the willingness of German General Practitioners to participate in primary care research and their attitude towards research in general practice. The results should enable a more successful approach to GPs in further studies.

**Methods:**

Cross sectional study using semi-structured interviews with a random sample of 76 General Practitioners who participate in the teaching of medical students at the University of Heidelberg.

**Results:**

Despite little experience, over 85 % of GPs appreciated research in their field. Important reasons for scepticism about research were the gap between theoretical research and practical work of GPs and the domination of research by specialists. Main barriers for participation are clinical workload, administrative overload and the newly introduced Disease Management Programs. The highest motivation for GPs to participate in research emanates from the will to substantiate their quality of care with solid research data.

**Conclusions:**

Financial incentives and personal support e.g. with study nurses are certainly necessary to establish a research culture and to overcome main barriers against participation. The most successful approach to motivate GPs to participate is to convince them that research documents their quality of care. This data may reflect the facts on which the financial resources are provided in the future health care system.

## Background

Compared to other European countries, Germany still has little of a research tradition in general practice. Increasingly policy-makers have realized that the continuity and the efficacy of the healthcare system have to be improved. For this a well-developed primary medical care system is needed. In recent years a number of new chairs of general practice have been established and a national funding programme was created in order to promote General Practice as an academic discipline in Germany. The University of Heidelberg, which is the oldest university in Germany, is among the beneficiaries of these developments. Despite of a well established network of teaching practices, the research group for general practice and health services research was only created in 2002 [1]. This group faces the challenge to perform studies with general practitioners who have little experience with participation in scientific research. It is known, that by specifically addressing strategies significant improvements in participation rates can be achieved [2]. The aim of the study was to investigate the willingness of GPs to participate in research and to learn about their attitude towards research in their field in general. These data should help to create successful approaches for further projects.

## Methods

### Study design

We performed a cross sectional observational study collecting qualitative data. The Ethical Commission of the University of Heidelberg approved the study.

### Study population

A random sample of 76 GPs in the area of Heidelberg was approached for the study. The GPs were selected by choosing every third of an alphabetical list of 250 practices. These GPs were associated with the university by frequently teaching students in their practices. Due to old data, in six cases the GPs did not practice any more. So finally 76 GPs were included. All of the selected GPs were in practice for more than five years. Former studies indicated that relevance of the topic has a positive predictive value for the recruitment rate. Therefore we selected a topic with a high clinical relevance in daily practice: osteoarthritis [3,4]. Based on this information we performed a fictitious study, aiming at improving the quality of care of patients with osteoarthritis. The GPs received an official letter from the Department of General Practice and Health Services Research. This letter contained detailed information about the relevance of the topic, the aim of the study and the possible benefit for GPs, their teams and their patients. They were also informed about the time requirement for the study, which was estimated to be 30 minutes. The allowance for participating was fixed to 50 Euro to exclude financial reasons for consent. The letter concluded with the request to fax an agreement form back to the university.

### Measures

No letters were returned to the university because of wrong addresses. A reminder or anything similar did not follow the first letter. One week after the letter, every GP was called by the principal investigator and was asked – after giving him again information on the study – if she or he wanted to participate. This approach was chosen to get qualitative information of all approached GPs about their willingness to participate and their opinion in general. This way of data collecting has already been used in this field of research and enables not only a high rate of data response, it is also a feasible way of collecting qualitative data [5]. If the GP decided not to participate, her or his reason to do so were recorded without further discussion. Every GP, whether he denied or agreed to participate was asked about his opinions concerning research in general practice in general and the relevance of the research topic to him or her. The GPs who agreed to participate where asked to fax the sheet of agreement.

### Analysis

We were mainly focused on qualitative information. Therefore the statements of the GPs were grouped and coded by two separate researchers and then discussed in order to agree on the selected categorisation according to the guidelines for qualitative researchers [6].

## Results

A total of 18 GPs (23.8%) of the approached GPs was female, 58 GPs (72.2 %) were male. Only two GPs faxed their agreement-sheet within the first week, before they were phoned and interviewed by the principal investigator. During the telephone calls 25 GPs (32.8 %) agreed to participate and promised to fax the sheet. Out of this group 5 GPs (18.5%) sent their fax during the subsequent two weeks. A total of 8 (10.5 %) faxes were returned. Five female (27.7 %) and 22 male GPs agreed (37.9 %) to participate. A total of 27 GPs agreed to participate ultimately.

Table 2 shows the GPs reasons for non-participation. 24 (31.5 %) of the GPs argued they had no time, because of overwork in their practice caused by the daily routine work. The second most frequent reason named was the regular administrative workload. Seven GPs specified this argument by blaming especially the newly introduced "disease management program, DMP", founded by German sick funds for chronic illnesses like diabetes and hypertension. This program was perceived to increase the daily paperwork tremendously. Other important reasons for non-participation were disbelief that possible results can be implemented in daily work without financial incentives. GPs argued that changes, which are accompanied by any additional time effort, could only be implemented in daily practice if they receive adequate financial reimbursement. "Money sets the course", as one GP stated. Two GPs declared they had no problem in dealing with osteoarthritis and regarded also dealing patients suffering from osteoarthritis quite easy. Four GPs named participation in courses and congresses as a reason for non-participating. One GP mentioned that this kind of research is only for academic interest and helps only the career of the researcher. An other GP argued that he already feels monitored by all the data collected by health insurance and the government.

**Table 2:** Reasons mentioned by GPs for non-participation in research

	n	%
Overwork in practice	24	31.6
Already too much paperwork / bureaucracy	13	17.1
The results might not be implemented in practice because of financial constrains	10	13.2
Overload because of "disease management program"	7	9.2
No belief in results because of the degenerative progress of the illness	5	6.6
Personal time exposure for courses, etc.	4	5.3
Private reasons	2	2.6
Adherence to an other study at the same time	2	2.6
To less connection between (theoretical) university research and practical work as a GP	2	2.6
No problem in treating arthritis patients	2	2.6
No decision	1	1.3
Feeling of being monitored	1	1.3
Only the researcher takes benefit out of this research	1	1.3
**Total**	**76**	**100**

As can be seen in table 3, 85.6 % of the GPs had positive attitudes regarding research in their field. They consider it reasonable and eligible, but in most of these cases the answer was not substantiated with a further argumentation. Interestingly, answers, which were allocated to the category „makes sense because it improves the reputation of GPs and documents our quality of care", were only given by GPs who agreed to participate in the study. So this aspect seemed to be the most important motivation for an GP to take part in research. In addition, this particular group of GPs regularly added further comments regarding role of the GP in the German health care system. Important reasons for scepticism were the gap between theoretical research and practical work and the domination of research by specialists. One GP argued it would be better to spend more money on treatment than on research.

**Table 3:** GPs' attitudes regarding research in General Practice in general

	n	%
Reasonable and eligible	54	71.1
Makes sense because it improves the reputation of GPs	11	14.5
Not sure if it makes sense ("I am not convinced"), no further explanation	3	3.9
University research and daily work in family medicine have only little in common	3	3.9
Makes no sense because research is dominated by specialists	2	2.6
Does not lead to results (without more explanation)	1	1.3
Better more money for the GPs then for research	1	1.3
Feeling of being monitored	1	1
**Total**	**76**	**100**

## Discussion

There were three main conclusions that can be drawn out of our interview results. Firstly, the research topic improving the quality of care for patients suffering from osteoarthritis was considered as highly relevant by the interviewed GPs. This is concordant to our assumptions based on epidemiological data, which led to the fictitious research topic. The same reasoning causes GPs to seek support in the daily treatment of patients with osteoarthritis. Consequently this will be subject of future research projects.

Secondly, most of the GPs appreciate research in general practice, but a few were very sceptical. German GPs still don't realise it as a professional obligation as their colleagues in countries like e.g. the Netherlands or the United Kingdom, with a much longer tradition in research, do [7].

The third main result of our survey has not yet been shown in former studies. It is the fact that the willingness for participating in research emanates mainly out of the motivation to improve the reputation of family medicine in general by documenting the high quality of care with data attained in solid surveys. This may reflect the increasing self-confidence of German GPs, which are about to expend the influence in the health care system, and their awareness that an own research culture helps to enhance this. Facing decreasing financial resources in the Health care system, GPs may also be aware that a solid database documenting the quality of care will get more important for the distribution of financial resources in the near future.

The revealed barriers against participating in studies mentioned in our telephone survey are in line with results from previous studies in other countries [8]. According to those former results, relevance of the research topic, reimbursement and compatibility with routine general practice work are important factors. Ideally the GPs are embedded in an existing research culture [7,9,10]. Study nurses or mentors could be an important factor to enhance GPs' preparedness to participate in General Practice research because they reduce the administrative workload for GPs and enhance the motivation to participate in research [8,10,11]. Furthermore financial incentives for participation are essential because of time constraints and overwhelming administrative work that compete with research and represent important barriers [8,11]. An unexpected quantitative result of this study was that being involved with the training of medical students and being linked with the University is not reflected per se in a higher motivation in participating in research. Participating rates of about 30 % are usually achieved in random postal mailings to GPs without academic affiliation [5,9,13,14]. Previous studies have shown that involvement in student teaching represents a positive predictive factor for participation in research, so we assumed to achieve a much higher participation rate. It appears that a well-established teaching network does not necessarily yield much benefit for research purposes [11].

## Conclusions

Previous studies were mainly focused on formal or external barriers for GPs against participating in research, or revealed approaches that cannot easily be transferred, as e.g. the enrollment of friendly GPs [15]. What this study adds is that there is an important target to aim at, if GPs have to be involved in research: the motivation to underline their daily work with solid data reflecting their high quality of care. With this knowledge GPs may be easier approached if they need to be motivated to participate in future projects. Aiming more on psychological targets, this approach should be transferable to other countries as well. However, researchers should be aware that beside the chance of motivating GPs, this strategy also contains a risk: GPs could be discouraged and kept away from future participation if the anticipated demonstration of their quality of care is not as obvious as expected.

## Competing interests

The author(s) declare that they have no competing interests.

## Authors' contributions

TR conceived and performed the study and draft the manuscript. JS participated in the study design. All authors read and approved the final manuscript.

**Table 1:** Agreement for participation related to sex

Sex	n	Agreement after letter	Agreement during telephone call	Total agreement
Male	58	2	20	22 (37.9%)
Female	18	0	5	5 (27.8%)
Total	76	2 (2.6 %)	25 (32.8 %)	27 (35.5 %)

## Pre-publication history

The pre-publication history for this paper can be accessed here:


